# Supplementary material for: Pine polyphenols from Pinus koraiensis prevent injuries induced by gamma radiation in mice
Source: PeerJ. 2016 Apr 5;4:e1870. doi: 10.7717/peerj.1870 (PMC4824883; doi:10.7717/peerj.1870)
Supplement: Table S1 — EC50 or RP0.5 of Antioxidant activities of PPs from Pinus koraiensis. [file peerj-04-1870-s001.doc]

**Supplemental Information**

**Table 1S.** EC50 or RP0.5 of antioxidant activities of PPs from *Pinus koraiensis*

|  | EC_50_ (μg/mL) | | | RP_0.5_ (μg/mL) |
| --- | --- | --- | --- | --- |
|  | DPPH· | O_2_^-·^ | OH· |  |
| Pine polyphenols | 324.0±13.52 | 117.1±6.13 | 519.9±25.91 | 363.2±5.34 |
| Catechin | < 100 | < 50 | 946.4±18.94 | < 100 |
| Gallic acid | < 100 | 65.9±3.17 | < 100 | < 100 |

Each value represents mean ± SD (n = 3).

EC_50_ (μg/mL) values are calculated from the regression lines using six different concentrations (50–1200 μg/mL) in triplicate and their data are presented as 50% scavenging activities.

RP_0.5_ values are presented by the sample concentrations at 0.5 of absorbance value at 700 nm.
