# Supplementary material for: Pine polyphenols from Pinus koraiensis prevent injuries induced by gamma radiation in mice
Source: PeerJ. 2016 Apr 5;4:e1870. doi: 10.7717/peerj.1870 (PMC4824883; doi:10.7717/peerj.1870)
Supplement: Data S1 — Raw data-Pine polyphenols restrains [file peerj-04-1870-s002.doc]

**RAW DATA**

| **Fig.1a.** | Mean | SE(±) | Mean | SE(±) | Mean | SE(±) |
| --- | --- | --- | --- | --- | --- | --- |
|  | DPPH free radical scavenging activity | | | | | |
| Concentration | PPs |  | Catechin |  | Gallic acid |  |
| 0.1 | 15.76 | 0.675 | 60.08 | 2.363 | 90.04 | 3.493 |
| 0.2 | 28.59 | 1.224 | 85.67 | 3.062 | 95.34 | 4.872 |
| 0.3 | 45.96 | 1.309 | 87.78 | 4.762 | 96.59 | 3.389 |
| 0.4 | 58.83 | 2.482 | 89.45 | 3.004 | 97.87 | 4.904 |
| 0.5 | 68.52 | 3.299 | 89.39 | 2.268 | 98.98 | 3.682 |
| 0.6 | 71.07 | 3.196 | 91.27 | 5.162 | 96.34 | 3.472 |
| **Fig.1b.** | superoxide anion radical scavenging activity | | | | | |
| Concentration | PPs |  | Catechin |  | Gallic acid |  |
| 0.05 | 12.88 | 1.478 | 54.05 | 2.685 | 47.14 | 3.382 |
| 0.1 | 21.77 | 0.956 | 63.87 | 2.832 | 66.17 | 2.678 |
| 0.15 | 49.09 | 1.238 | 66.39 | 3.674 | 75.16 | 2.169 |
| 0.2 | 64.27 | 2.542 | 77.75 | 2.564 | 86.18 | 3.134 |
| 0.25 | 83.80 | 3.450 | 78.85 | 1.457 | 97.19 | 2.467 |
| 0.3 | 89.39 | 4.425 | 78.79 | 3.321 | 98.13 | 3.984 |
| **Fig.1c.** | hydroxyl radical scavenging activity | | | | | |
| Concentration | PPs |  | Catechin |  | Gallic acid |  |
| 0.2 | 22.70 | 4.663 | 15.03 | 3.416 | 53.35 | 1.876 |
| 0.4 | 39.40 | 3.327 | 25.03 | 3.216 | 60.14 | 1.603 |
| 0.6 | 51.10 | 4.990 | 36.18 | 2.968 | 62.194 | 1.520 |
| 0.8 | 58.79 | 2.653 | 48.10 | 2.489 | 63.732 | 1.458 |
| 1 | 71.68 | 4.474 | 52.97 | 2.293 | 64.885 | 1.412 |
| 1.2 | 72.56 | 4.295 | 55.22 | 0.037 | 68.885 | 1.698 |
| **Fig.1d.** | reducing power assay | | | | | |
| Concentration | PPs |  | Catechin |  | Gallic acid |  |
| 0.1 | 0.215 | 0.00283 | 0.577 | 0.0173 | 2.048 | 0.0267 |
| 0.2 | 0.371 | 0.00707 | 1.039 | 0.0300 | 2.490 | 0.0319 |
| 0.3 | 0.393 | 0.00283 | 1.435 | 0.0404 | 2.468 | 0.0478 |
| 0.4 | 0.552 | 0.00707 | 1.803 | 0.0917 | 2.393 | 0.0887 |
| 0.5 | 0.663 | 0.0120 | 2.134 | 0.0896 | 2.486 | 0.0969 |
| 0.6 | 0.770 | 0.0134 | 2.298 | 0.0983 | 2.444 | 0.0954 |

| **Fig.2.** |  | | |
| --- | --- | --- | --- |
| Groups | Mean | SE(±) | *P* |
| Control | 2.87 | 0.326 |  |
| IR | 1.30 | 0.125 |  |
| 50 (PPs + IR) | 1.52 | 0.290 | ^*^ < 0.05 |
| 100 (PPs + IR) | 1.65 | 0.206 | ^*^ < 0.05 |
| 200 (PPs + IR) | 1.93 | 0.257 | ^*^ < 0.05 |

| **Fig.3.** |  | | |
| --- | --- | --- | --- |
| MDA (Serum) |  | | |
| Groups | Mean | SE(±) | *P* |
| Control | 27.81 | 3.015 |  |
| PPs | 28.13 | 3.242 |  |
| IR | 47.11 | 1.836 | ^*^ < 0.05 |
| PPs + IR | 32.04 | 1.463 | ^#^ < 0.05 |
| MDA (Liver) |  | | |
| Groups | Mean | SE(±) | *P* |
| Control | 1.98 | 0.181 |  |
| PPs | 1.86 | 0.084 |  |
| IR | 4.94 | 0.242 | ^*^ < 0.05 |
| PPs + IR | 2.65 | 0.053 | ^*^ < 0.05, ^#^ < 0.05 |
| MDA (Spleen) |  | | |
| Groups | Mean | SE(±) | *P* |
| Control | 2.96 | 0.113 |  |
| PPs | 2.89 | 0.086 |  |
| IR | 6.72 | 0.192 | ^*^ < 0.05 |
| PPs + IR | 3.84 | 0.335 | ^*^ < 0.05, # < 0.05 |

| **Fig.4a.** |  | | |
| --- | --- | --- | --- |
| SOD (Serum) |  | | |
| Groups | Mean | SE(±) | *P* |
| Control | 119.05 | 6.723 |  |
| PPs | 113.14 | 8.966 |  |
| IR | 79.55 | 3.891 | ^*^ < 0.05 |
| PPs + IR | 88.28 | 7.363 | ^*^ < 0.05 |
| SOD (Liver) |  | | |
| Groups | Mean | SE(±) | *P* |
| Control | 120.19 | 10.152 |  |
| PPs | 108.49 | 7.163 |  |
| IR | 81.19 | 2.517 | ^*^ < 0.05 |
| PPs + IR | 92.01 | 4.381 | ^*^ < 0.05 |
| SOD (Spleen) |  | | |
| Groups | Mean | SE(±) | *P* |
| Control | 97.99 | 3.091 |  |
| PPs | 97.68 | 9.563 |  |
| IR | 17.65 | 0.975 | ^*^ < 0.05 |
| PPs + IR | 24.71 | 8.113 | ^*^ < 0.05 |
| **Fig.4b.** |  | | |
| CAT (Serum) |  | | |
| Groups | Mean | SE(±) | *P* |
| Control | 10.13 | 0.292 |  |
| PPs | 9.55 | 1.282 |  |
| IR | 4.29 | 0.514 | ^*^ < 0.05 |
| PPs + IR | 7.91 | 0.627 | ^#^ < 0.05 |
| CAT (Liver) |  | | |
| Groups | Mean | SE(±) | *P* |
| Control | 16.05 | 1.313 |  |
| PPs | 15.11 | 0.041 |  |
| IR | 11.98 | 0.542 | ^*^ < 0.05 |
| PPs + IR | 15.23 | 1.493 | ^#^ < 0.05 |
| CAT (Spleen) |  | | |
| Groups | Mean | SE(±) | *P* |
| Control | 8.79 | 0.044 |  |
| PPs | 8.98 | 0.113 |  |
| IR | 3.16 | 0.073 | ^*^ < 0.05 |
| PPs + IR | 7.34 | 0.447 | ^#^ < 0.05 |

| **Fig.4c.** |  | | |
| --- | --- | --- | --- |
| GSH-Px (Serum) |  | | |
| Groups | Mean | SE(±) | *P* |
| Control | 133.12 | 7.744 |  |
| PPs | 130.22 | 13.91 |  |
| IR | 54.98 | 5.013 | ^*^ < 0.05 |
| PPs + IR | 104.69 | 7.916 | ^*^ < 0.05, ^#^ < 0.05 |
| GSH-Px (Liver) |  | | |
| Groups | Mean | SE(±) | *P* |
| Control | 79.56 | 2.253 |  |
| PPs | 80.01 | 6.992 |  |
| IR | 42.19 | 3.746 | ^*^ < 0.05 |
| PPs + IR | 63.84 | 3.641 | ^*^ < 0.05, ^#^ < 0.05 |
| GSH-Px (Spleen) |  | | |
| Groups | Mean | SE(±) | *P* |
| Control | 76.26 | 6.843 |  |
| PPs | 72.87 | 4.657 |  |
| IR | 49.66 | 3.753 | ^*^ < 0.05 |
| PPs + IR | 62.15 | 4.041 |  |
| **Fig.4d.** |  | | |
| GSH (Serum) |  | | |
| Groups | Mean | SE(±) | *P* |
| Control | 22.69 | 1.188 |  |
| PPs | 23.28 | 2.362 |  |
| IR | 6.19 | 1.043 | ^*^ < 0.05 |
| PPs + IR | 15.62 | 2.044 | ^*^ < 0.05, ^#^ < 0.05 |
| GSH (Liver) |  | | |
| Groups | Mean | SE(±) | *P* |
| Control | 26.79 | 2.272 |  |
| PPs | 24.62 | 2.491 |  |
| IR | 19.48 | 1.634 | ^*^ < 0.05 |
| PPs + IR | 22.51 | 0.278 |  |
| GSH (Spleen) |  | | |
| Groups | Mean | SE(±) | *P* |
| Control | 34.76 | 2.578 |  |
| PPs | 33.51 | 0.891 |  |
| IR | 26.82 | 1.412 | ^*^ < 0.05 |
| PPs + IR | 28.78 | 1.636 | ^*^ < 0.05 |

| **Fig.6b.** |  | | |
| --- | --- | --- | --- |
| Bcl-2 expression |  | | |
| Groups | Mean | SE(±) | *P* |
| Control | 1.00 | 0.135 |  |
| IR | 0.54 | 0.036 | ^*^ < 0.05 |
| PPs | 0.93 | 0.097 |  |
| PPs + IR | 0.72 | 0.033 | ^*^ < 0.05, ^#^ < 0.05 |
| **Fig.6c.** |  | | |
| Bax expression |  | | |
| Groups | Mean | SE(±) | *P* |
| Control | 1.00 | 0.115 |  |
| IR | 2.57 | 0.174 | ^*^ < 0.05 |
| PPs | 1.19 | 0.079 |  |
| PPs + IR | 1.94 | 0.143 | ^*^ < 0.05, ^#^ < 0.05 |
| **Fig.6d.** |  | | |
| Bax/Bcl-2 |  | | |
| Groups | Mean | SE(±) | *P* |
| Control | 1.00 | 0.108 |  |
| IR | 4.76 | 0.374 | ^*^ < 0.05 |
| PPs | 1.27 | 0.137 |  |
| PPs + IR | 2.70 | 0.203 | ^*^ < 0.05, ^#^ < 0.05 |
| **Fig.6e.** |  | | |
| [Cytochrome](http://dict.youdao.com/w/cytochrome/) [c](http://dict.youdao.com/w/c/) expression |  | | |
| Groups | Mean | SE(±) | *P* |
| Control | 1.00 | 0.097 |  |
| IR | 4.41 | 0.298 | ^*^ < 0.05 |
| PPs | 1.06 | 0.096 |  |
| PPs + IR | 2.84 | 0.233 | ^*^ < 0.05, ^#^ < 0.05 |
| **Fig.6f.** |  | | |
| Caspase-3 expression |  | | |
| Groups | Mean | SE(±) | *P* |
| Control | 1.00 | 0.121 |  |
| IR | 3.65 | 0.246 | ^*^ < 0.05 |
| PPs | 0.92 | 0.089 |  |
| PPs + IR | 2.01 | 0.136 | ^*^ < 0.05, ^#^ < 0.05 |

* represents value significantly different from the Control group (*P* < 0.05).

# represents value significantly different from the IR group (*P* < 0.05).
